# Longitudinal timepoint assessment of patient-reported clinical disease activity in ulcerative colitis and Crohn’s disease: suitability of a rapid Patient-Reported Outcome 2 (PRO-2) assessment

**DOI:** 10.1093/ecco-jcc/jjag153

**Published:** 2026-11-03

**Authors:** Lauren C Beck, Gregory R Young, Nicola J Wyatt, Robert J Mulligan, Robert D Lees, Chaonan Dong, Hannah Watson, Trevor Liddle, Ally Speight, Dean M Allerton, Victoria Hildreth, Naomi S McGregor, Ruth Wood, Mary Doona, Debbie Alexander, Samantha Baillie, Biljana Brezina, Beatriz Camesella Perez, Bessie Cipriano, Jessica Cordle, Dominic Devine, Alexander T Elford, Kay Goulden, Marie Green, Hannah Husband, Lorna Ingoe, Lauren John, Sunita Dias Juliao, Andrew King, Klaartje Kok, Yasmin Laidler, Isabel Laszczak, Sarah Lawrence, Irish Lee, Melanie Love, Julian Marchesi, Claire McQuillian, Helen Millage, Ibinye Minimah, Alison Moss, Felicia Onoviran, Christopher Palmer-Jones, Gareth C Parkes, Sally Payton, Jahleesha Pinney, Kiran Prabhu, Chandni Radia, Parizade Raymode, Juan de la Revilla Negro, Francesca Rosa, Lydia Scarlett, Mohammed T Sharip, Natalie Shields, Sreedhar Subramanian, Robert Varley, Busiswa Vuyelele-Titchmarsh, Valentina Toska, Kevin Whelan, Kate Wistance, Mark Yongco, Lisa Younge, Nick Powell, Johanne Brooks-Warburton, Rachel Cooney, Simon P Borg-Bartolo, Shaji Sebastian, Benjamin Disney, Jack J Satsangi, Alan Steel, Konrad Koss, James C Lee, Anjan Dhar, Dharmaraj Durai, Philip Harvey, Christian P Selinger, Alexandra J Kent, Jeffrey R Butterworth, Jimmy K Limdi, Melissa A Smith, Peter M Irving, Ajay M Verma, Ailsa L Hart, Emma Wesley, Ian L P Beales, Jiezel De Guzman, Praveen Rajasekhar, Tariq Ahmad, Jonathan Blackwell, Charlie W Lees, Fraser Cummings, Kamal V Patel, Miles Parkes, Christopher J Stewart, Luke Jostins-Dean, James O Lindsay, Vipul Jairath, Nicholas A Kennedy, Tim Raine, IBD-RESPONSE collaborators, Christopher A Lamb

**Affiliations:** Faculty of Medical Sciences, Translational and Clinical Research Institute, Newcastle University, Newcastle upon Tyne, United Kingdom; Faculty of Medical Sciences, Translational and Clinical Research Institute, Newcastle University, Newcastle upon Tyne, United Kingdom; Faculty of Medical Sciences, Translational and Clinical Research Institute, Newcastle University, Newcastle upon Tyne, United Kingdom; The Newcastle upon Tyne Hospitals NHS Foundation Trust, Newcastle upon Tyne, United Kingdom; Faculty of Medical Sciences, Translational and Clinical Research Institute, Newcastle University, Newcastle upon Tyne, United Kingdom; The Newcastle upon Tyne Hospitals NHS Foundation Trust, Newcastle upon Tyne, United Kingdom; Faculty of Medical Sciences, Translational and Clinical Research Institute, Newcastle University, Newcastle upon Tyne, United Kingdom; The Newcastle upon Tyne Hospitals NHS Foundation Trust, Newcastle upon Tyne, United Kingdom; Faculty of Medical Sciences, Translational and Clinical Research Institute, Newcastle University, Newcastle upon Tyne, United Kingdom; The Newcastle upon Tyne Hospitals NHS Foundation Trust, Newcastle upon Tyne, United Kingdom; Faculty of Medical Sciences, Translational and Clinical Research Institute, Newcastle University, Newcastle upon Tyne, United Kingdom; The Newcastle upon Tyne Hospitals NHS Foundation Trust, Newcastle upon Tyne, United Kingdom; Faculty of Medical Sciences, Translational and Clinical Research Institute, Newcastle University, Newcastle upon Tyne, United Kingdom; The Newcastle upon Tyne Hospitals NHS Foundation Trust, Newcastle upon Tyne, United Kingdom; Newcastle Clinical Trials Unit, Newcastle University, Newcastle upon Tyne, United Kingdom; Newcastle Clinical Trials Unit, Newcastle University, Newcastle upon Tyne, United Kingdom; Newcastle Clinical Trials Unit, Newcastle University, Newcastle upon Tyne, United Kingdom; Newcastle Clinical Trials Unit, Newcastle University, Newcastle upon Tyne, United Kingdom; The Newcastle upon Tyne Hospitals NHS Foundation Trust, Newcastle upon Tyne, United Kingdom; The Edinburgh IBD Unit, Western General Hospital, Edinburgh, United Kingdom; Institute of Genetics and Cancer, University of Edinburgh, Edinburgh, United Kingdom; St George’s University Hospitals NHS Foundation Trust, London, United Kingdom; Cambridge University Hospitals NHS Trust, Cambridge, United Kingdom; Oxford University Hospitals NHS Foundation Trust, Oxford, United Kingdom; Barts Health NHS Trust, London, United Kingdom; Guy’s and St Thomas’ NHS Foundation Trust, London, United Kingdom; Northumbria Healthcare NHS Foundation Trust, North Tyneside, United Kingdom; The Edinburgh IBD Unit, Western General Hospital, Edinburgh, United Kingdom; Northern Care Alliance NHS Foundation Trust, Greater Manchester, United Kingdom; The Royal Wolverhampton NHS Trust, Wolverhampton, United Kingdom; Royal Devon University Healthcare NHS Foundation Trust, Exeter, United Kingdom; Northumbria Healthcare NHS Foundation Trust, North Tyneside, United Kingdom; Royal Devon University Healthcare NHS Foundation Trust, Exeter, United Kingdom; The Royal Wolverhampton NHS Trust, Wolverhampton, United Kingdom; The Newcastle upon Tyne Hospitals NHS Foundation Trust, Newcastle upon Tyne, United Kingdom; Barts Health NHS Trust, London, United Kingdom; The Newcastle upon Tyne Hospitals NHS Foundation Trust, Newcastle upon Tyne, United Kingdom; Imperial College NHS Foundation Trust, London, United Kingdom; Newcastle Clinical Trials Unit, Newcastle University, Newcastle upon Tyne, United Kingdom; Barts Health NHS Trust, London, United Kingdom; University Hospitals Birmingham NHS Foundation Trust, Birmingham, United Kingdom; Department of Metabolism Digestion and Reproduction, Imperial College London, London, United Kingdom; Institute of Genetics and Cancer, University of Edinburgh, Edinburgh, United Kingdom; University Hospitals Coventry and Warwickshire NHS Foundation Trust, Coventry, United Kingdom; Royal Devon University Healthcare NHS Foundation Trust, Exeter, United Kingdom; Somerset NHS Foundation Trust, Taunton, United Kingdom; Leeds Teaching Hospitals NHS Trust, Leeds, United Kingdom; Royal Free London NHS Foundation Trust, London, United Kingdom; Barts Health NHS Trust, London, United Kingdom; Royal Devon University Healthcare NHS Foundation Trust, Exeter, United Kingdom; Northern Care Alliance NHS Foundation Trust, Greater Manchester, United Kingdom; University Hospital Southampton NHS Foundation Trust, Southampton, United Kingdom; King’s College Hospital NHS Foundation Trust, London, United Kingdom; Kettering General Hospital NHS Foundation Trust, Kettering, United Kingdom; Cambridge University Hospitals NHS Trust, Cambridge, United Kingdom; Cambridge University Hospitals NHS Trust, Cambridge, United Kingdom; University Hospital Southampton NHS Foundation Trust, Southampton, United Kingdom; Cambridge University Hospitals NHS Trust, Cambridge, United Kingdom; St George’s University Hospitals NHS Foundation Trust, London, United Kingdom; Cambridge University Hospitals NHS Trust, Cambridge, United Kingdom; Royal Free London NHS Foundation Trust, London, United Kingdom; Cambridge University Hospitals NHS Trust, Cambridge, United Kingdom; University Hospitals Sussex NHS Foundation Trust, Brighton, United Kingdom; Department of Nutritional Sciences, King’s College London, London, United Kingdom; The Shrewsbury and Telford Hospital NHS Trust, Shrewsbury, United Kingdom; Barts Health NHS Trust, London, United Kingdom; St Mark’s Hospital, London, United Kingdom; Imperial College NHS Foundation Trust, London, United Kingdom; Department of Metabolism Digestion and Reproduction, Imperial College London, London, United Kingdom; East and North Hertfordshire Teaching NHS Trust, Stevenage, United Kingdom; University Hospitals Birmingham NHS Foundation Trust, Birmingham, United Kingdom; College of Medicine and Health, University of Birmingham, West Midlands, United Kingdom; Manchester University NHS Foundation Trust, Manchester, United Kingdom; Hull University Teaching Hospitals NHS Trust, Hull, United Kingdom; Clinical Sciences Centre, Hull York Medical School, Hull, United Kingdom; University Hospitals Coventry and Warwickshire NHS Foundation Trust, Coventry, United Kingdom; Oxford University Hospitals NHS Foundation Trust, Oxford, United Kingdom; Translational Gastroenterology and Liver Unit, University of Oxford, Oxford, United Kingdom; Liverpool University Hospitals NHS Foundation Trust, Liverpool, United Kingdom; East Cheshire NHS Trust, Macclesfield, United Kingdom; King’s College Hospital NHS Foundation Trust, London, United Kingdom; Institute for Liver and Digestive Health, University College London, London, United Kingdom; Genetic Mechanisms of Disease Laboratory, The Francis Crick Institute, London, United Kingdom; County Durham and Darlington NHS Foundation Trust, Darlington, United Kingdom; School of Health and Life Sciences, Teeside University, Middlesbrough, United Kingdom; Cardiff and Vale University Health Board, Cardiff, United Kingdom; The Royal Wolverhampton NHS Trust, Wolverhampton, United Kingdom; Leeds Teaching Hospitals NHS Trust, Leeds, United Kingdom; King’s College Hospital NHS Foundation Trust, London, United Kingdom; The Shrewsbury and Telford Hospital NHS Trust, Shrewsbury, United Kingdom; Northern Care Alliance NHS Foundation Trust, Greater Manchester, United Kingdom; Manchester Academic Health Sciences Department of Biology, University of Manchester, Manchester, United Kingdom; University Hospitals Sussex NHS Foundation Trust, Brighton, United Kingdom; Department of Medical Education, Brighton and Sussex Medical School, Brighton, United Kingdom; Guy’s and St Thomas’ NHS Foundation Trust, London, United Kingdom; Peter Gorer Department of Immunobiology, King’s College London, London, United Kingdom; Kettering General Hospital NHS Foundation Trust, Kettering, United Kingdom; Department of Metabolism Digestion and Reproduction, Imperial College London, London, United Kingdom; St Mark’s Hospital, London, United Kingdom; Somerset NHS Foundation Trust, Taunton, United Kingdom; Norfolk and Norwich University Hospitals NHS Foundation Trust, Norwich, United Kingdom; Norfolk and Norwich University Hospitals NHS Foundation Trust, Norwich, United Kingdom; Northumbria Healthcare NHS Foundation Trust, North Tyneside, United Kingdom; Royal Devon University Healthcare NHS Foundation Trust, Exeter, United Kingdom; Exeter Inflammatory Bowel Disease and Pharmacogenetics Research Group, University of Exeter, Exeter, United Kingdom; The Edinburgh IBD Unit, Western General Hospital, Edinburgh, United Kingdom; Institute of Genetics and Cancer, University of Edinburgh, Edinburgh, United Kingdom; The Edinburgh IBD Unit, Western General Hospital, Edinburgh, United Kingdom; Institute of Genetics and Cancer, University of Edinburgh, Edinburgh, United Kingdom; University Hospital Southampton NHS Foundation Trust, Southampton, United Kingdom; Faculty of Medicine, University of Southampton, Southampton, United Kingdom; St George’s University Hospitals NHS Foundation Trust, London, United Kingdom; Cambridge University Hospitals NHS Trust, Cambridge, United Kingdom; Faculty of Medical Sciences, Translational and Clinical Research Institute, Newcastle University, Newcastle upon Tyne, United Kingdom; Kennedy Institute of Rheumatology, University of Oxford, Oxford, United Kingdom; Barts Health NHS Trust, London, United Kingdom; London School of Medicine and Dentistry, Queen Mary University of London, London, United Kingdom; London Health Sciences Centre, Western University, London, ON, Canada; Royal Devon University Healthcare NHS Foundation Trust, Exeter, United Kingdom; Exeter Inflammatory Bowel Disease and Pharmacogenetics Research Group, University of Exeter, Exeter, United Kingdom; Cambridge University Hospitals NHS Trust, Cambridge, United Kingdom; Faculty of Medical Sciences, Translational and Clinical Research Institute, Newcastle University, Newcastle upon Tyne, United Kingdom; The Newcastle upon Tyne Hospitals NHS Foundation Trust, Newcastle upon Tyne, United Kingdom

**Keywords:** patient-reported outcomes, PRO-2, Crohn’s disease, ulcerative colitis

## Abstract

**Background & Aims:**

Patient-reported outcomes (PRO) are essential for monitoring disease activity and treatment response in inflammatory bowel disease (IBD). The conventional PRO-2 requires several days of prospective symptom recording, increasing participant burden and attrition in longitudinal research. We evaluated whether a “rapid PRO-2”, using 2 days of data collected retrospectively and prospectively in a single day, could serve as a reliable and pragmatic alternative to a 4-day PRO-2.

**Methods:**

Using data from the ongoing IBD-RESPONSE cohort (*n* = 684 Crohn’s disease [CD]; *n* = 548 ulcerative colitis [UC]), we compared 4-day PRO-2 and rapid PRO-2 scores at baseline and 14 weeks post-therapy to assess disease activity, clinical (non) response and remission.

**Results:**

At baseline, 87.1% (596/684) of CD and 88.7% (486/548) of UC patients remained in the same active/inactive disease activity category (Cohen’s kappa: CD 0.715; UC 0.762; both *P* < .001). At week 14, 77% (CD; 195/252) and 83.1% (UC; 193/232) remained in the same non-response/response/remission category (Cohen’s kappa: CD 0.653; UC 0.743; both *P* < .001). Discrepancies mainly involved adjacent category shifts (ie, between response and remission, or non-response and response), with minimal re-classification between remission and non-response (*n* = 7 CD and *n* = 2 UC). Greater symptom variability across the 4 days increased the likelihood of switching between remission and non-response (odds ratio [95% confidence interval], CD 5.0 [3.65–6.85]; UC 7.8 [5.6–11.0], both *P* < .001). Responsiveness to clinical change was comparable between instruments.

**Conclusions:**

The rapid PRO-2 retains essential symptom data and demonstrates strong agreement with the 4-day PRO-2, offering an alternative in longitudinal studies to minimize burden and attrition.

## 1. Introduction

Precision medicine approaches in inflammatory bowel disease (IBD) aim to tailor therapies based on individual patient characteristics, necessitating large-scale studies with robust, longitudinal data to identify biomarkers, develop predictive models, and assess treatment responses.^1^^,^^2^

A key component of assessing response to treatment in IBD research is the use of patient-reported disease activity indices, such as the 2-item patient-reported outcome (PRO-2), measuring stool frequency and abdominal pain in Crohn’s disease (CD) or stool frequency and rectal bleeding in ulcerative colitis (UC).^3-5^ These measures are essential for capturing symptom change over time to determine outcomes such as clinical response, non-response, and remission. While integral to clinical research these scores typically require several days of data collection at 2 or more longitudinal timepoints throughout a study. This extended data collection period can be a barrier to patient participation in research and may increase the risk of “questionnaire fatigue,” impacting completion rates and therefore lowering data quality. The resulting loss to follow up^6^^,^^7^ may be a particular problem where patients are asked to complete questionnaires over several days across multiple time-points. As part of many real-world IBD studies it is imperative that symptoms of flare are captured quickly to ensure that assessment does not delay patient treatment. Such information is especially important where studies include oral agents such as corticosteroids or Janus kinase inhibitor (JAKi) small molecule drugs that can be provided to patients from clinic and commenced quickly. These agents may also have a rapid onset of symptom improvement,^8-11^ therefore it is important that measures such as the PRO-2 accurately capture disease activity within a critical time window.

The PRO-2 score for CD was originally described using a 7-day symptom diary,^4^ but implementation across studies varies. While a 7-day diary remains a reference standard, real-world studies often handle missing data in different ways (ie, up to 4 days in Hoekman et al.), highlighting the heterogeneity in PRO-2 usage. A further consideration is that the original weighted PRO-2, where components were assigned arbitrary physician-derived weights, has been criticized for lacking patient-based validation. The FDA therefore favors the unweighted version.

The aim of IBD-RESPONSE is to define predictors of responsiveness to advanced therapies in CD and UC. It is a prospective, multicenter, observational study that aims to explore multi-omic predictors of treatment outcomes in IBD patients starting biologics or targeted small molecule therapies (JAKi or sphingosine-1-phosphate (S1P) receptor modulators).^1^ As part of IBD-RESPONSE, 4-day symptom diaries are collected to calculate PRO-2 scores prior to therapy commencement, and again 14 weeks after therapy starts. These are used to determine clinical response, clinical remission and non-response. To further explore streamlined approaches to symptom reporting, IBD-RESPONSE also incorporates a 1-day retrospective symptom recall on the first day of diary completion.

This study aims to undertake a methodological evaluation of whether a condensed 2-day symptom diary collected in a single day (retrospective day + first day of diary, termed the “rapid PRO-2”) can serve as a viable alternative to a 4-day prospective diary (termed the “4-day PRO-2”). By evaluating this approach, we aim to support future studies in IBD by providing an evidence base to reduce participant burden, in turn supporting participant inclusion and retention while maintaining data accuracy.

## 2. Methods

### Participants

As part of the ongoing IBD-RESPONSE study we consented 1271 adult patients with an existing diagnosis of IBD (CD, UC, or IBD-unclassified) who were planning to commence a new advanced therapy. Within the UC cohort, 19 patients were classified as IBD-unclassified. This study represents an exploratory cross-sectional methodological analysis of data accrued to January 2025 within the ongoing IBD-RESPONSE cohort. This analysis is independent of the prespecified primary and secondary outcome measures for IBD-RESPONSE. Recruitment to IBD-RESPONSE is contingent upon patients having “clinically active disease.” Patients are consented to take part prior to completing their PRO-2 diary. Patients that did not meet the PRO-2 threshold for “clinically active disease” after providing consent (as per Table 3 definitions), were not followed up in IBD-RESPONSE, but their baseline data is suitable for this standalone cross-sectional evaluation of PRO-2 measurement properties independent of study endpoints. Demographic and baseline PRO-2 data were retained for these participants who we present as clinically “not active” within this manuscript.

### Ethics

Ethical approval was obtained from the Wales Research Ethics Committee 5 (ref: 21/WA/0228). All participants provided informed written consent prior to taking part in the study. Consent forms were signed by participants remotely via electronic consent forms embedded into a study-specific REDCap (Research Electronic Data Capture) online database^12^ or on paper. Participants accessed eConsent forms remotely using a web browser on a personal laptop, tablet or mobile device. Valid consent was verified by an appropriately delegated member of the research team prior to research participation.

### PRO-2 questionnaires

Patients were asked to fill out a PRO-2 questionnaire detailing their IBD symptoms at both baseline and week 14 across a 4-day diary (Figure 1). These questionnaires have been adapted from the 7-day PRO-2 diaries developed for the assessment of UC and CD activity.^3-5^ For CD (Table 1), questions consisted of the number of liquid stools per day (continuous variable) and abdominal pain (0 = none, 1 = mild, 2 = moderate, and 3 = severe). For UC (Table 2), questions consisted of a stool frequency score and a rectal bleeding score. A total PRO-2 score was also calculated for UC patients, summing the average stool frequency and rectal bleeding scores before rounding to the nearest whole number. A minimum of 2 days’ data entry was required to calculate disease activity (at baseline) or non-response/response/remission (at week 14; Table 3).^1^ For the purpose of this study, the focus was on PRO-2 criteria, and we therefore did not define patients as non-response/response/remission at week 14 based on additional criteria such as resectional bowel surgery since baseline, use of oral corticosteroids at week 14 or failure to wean oral corticosteroids prescribed at baseline, as used in IBD-RESPONSE.^1^

**Figure 1.**
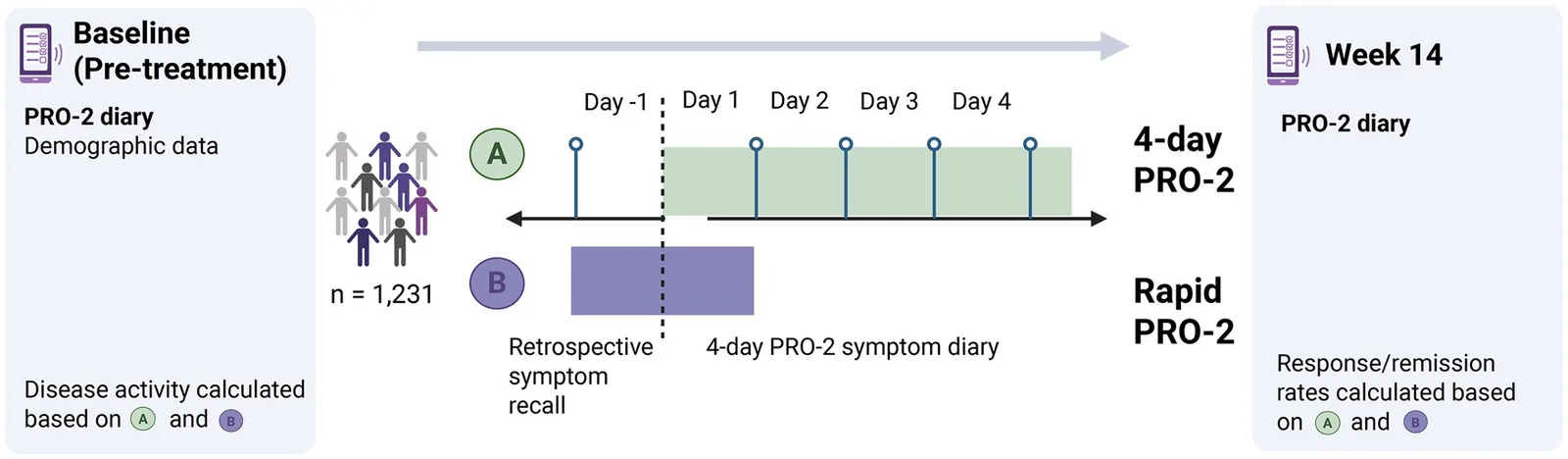
Overview of patient-reported symptom collection in IBD-RESPONSE and methodology for calculation of PRO-2 scores. Patients record a 4-day PRO-2 symptom diary, and a retrospective symptom recall. The 4-day PRO-2 is based on day 1-4 of the diary, while the rapid PRO-2 is based on the retrospective symptom recall, plus day 1 of the diary. IBD, inflammatory bowel disease; PRO, patient-reported outcomes.

**Table 1.** Unweighted PRO-2 calculation for CD for both the 4-day PRO-2 instrument and the rapid PRO-2 instrument.

Variable	Day prior to starting questionnaire (retrospective)	Day 1	Day 2	Day 3	Day 4	4-day PRO-2 mean (days 1-4)	Rapid PRO-2 mean (day prior + day 1)
**Number of liquid or very soft stools (SF)**							
**Abdominal pain (AP)** **0 = none** **1 = mild** **2 = moderate** **3 = severe**							

For unweighted PRO-2 CD calculation, a mean score is calculated for SF and AP. There is no rounding of mean data, no weighting of scores and no summing of scores to calculate a “total.”

Abbreviations: CD, Crohn’s disease; PRO, patient-reported outcomes; SF, stool frequency.

**Table 2.** PRO-2 calculation for UC, for both the 4-day PRO-2 instrument and the rapid PRO-2 instrument.

Variable	Day prior to starting questionnaire (retrospective)	Day 1	Day 2	Day 3	Day 4	4-day PRO-2 mean (days 1-4)	Rapid PRO-2 mean (day prior + day 1)
**Stool frequency (SF) score** **0 = Normal** **1 = 1-2 stools/day more than normal** **2 = 3-4 stools/day more than normal** **3 = 5 or more stools/day more than normal**							
**Rectal bleeding (RB) score** **0 = None** **1 = Visible blood with stool less than half the time** **2 = Visible blood with stool half of the time or more** **3 = Passing blood alone (requires patients to have ≥ 50% of bowel movements with visible blood AND ≥ 1 bowel movement with blood alone)**							
**PRO-2 UC (total):**						________________	________________

For PRO-2 UC calculation (total PRO-2), a mean is calculated for SF and RB, rounded to the nearest whole number (eg, 2.4 is rounded to 2; 2.6 is rounded to 3; 3.5 is rounded to 4), and added together.

Abbreviations: PRO, patient-reported outcomes; UC, ulcerative colitis.

**Table 3.** IBD-RESPONSE key PRO-2 definitions and thresholds for disease activity, clinical response, clinical remission, and non-response.

	CD	UC
**Clinically active disease (baseline)**	Stool frequency score of ≥4 and/or an abdominal pain score of ≥2	Total PRO-2 of ≥3 including a rectal bleeding score of ≥1
**Clinical response (week 14) in those with clinically active disease at baseline**	≥30% decrease in stool frequency score and/or abdominal pain score, and neither worse than baseline	Total PRO-2 decrease by ≥1 and ≥30% from baseline, and rectal bleeding score decrease by ≥1 or is ≤1
**Clinical remission (week 14) in those with clinically active disease at baseline**	Stool frequency score ≤2.8, abdominal pain score ≤1 and neither score worse than baseline	Stool frequency score of ≤1 and ≥1 decrease from baseline, and rectal bleeding score = 0
**Non-response (week 14) in those with clinically active disease at baseline**	Not meeting clinical response or remission criteria at week 14 assessment	Not meeting clinical response or remission criteria at week 14 assessment

Abbreviations: CD, Crohn’s disease; PRO, patient-reported outcomes; UC, ulcerative colitis.

### Statistical analysis

Analysis was performed in R v4.1.3.^13^ The ggsankey package v0.0.99999 was used to generate alluvial plots. Cohen’s kappa^14^ test was performed using the fmsb package v0.7.6 to assess agreement between the 2 scoring methods: rapid PRO-2 and 4-day PRO-2. Binomial logistic regression models were fit to the data to study the association between the variation in individual components of the PRO-2 questionnaires (based on standard deviation [σ]) and a change in disease activity between scoring methods at baseline. Cases where there was no change between scoring methods were coded as a 0, and cases where there was a change were coded as a 1. Model formulas included stool frequency and abdominal pain scores for CD, and stool frequency and rectal bleeding for UC. Adjusted odds ratios (ORs) with 95% confidence intervals (CIs) were calculated from the regression coefficients by exponentiation of the estimates.

Responsiveness to clinical change of the rapid PRO-2 and 4-day PRO-2 was assessed using the standardized response mean (SRM) for each individual item for CD and UC, and total PRO-2 for UC only. SRMs were calculated as the mean change from baseline to week 14 divided by the standard deviation of the change. Negative SRM values indicate improvement in symptoms, while positive values indicate worsening between baseline and week 14. Adjusted SRMs were calculated to account for the correlation between baseline and week 14 scores within individuals, allowing for interpretation using Cohen’s thresholds^15^:


$$\mathrm{Adjusted}\;\mathrm{effect}\;\mathrm{size}=\frac{\mathrm{SRM}}{\sqrt{2\;x\;(1-r)}},$$

where *r* is the Pearson correlation between baseline and week 14 scores. Pearson’s correlation was used, thereby treating ordinal PRO-2 items as continuous, to maintain comparability with published methods. As a sensitivity analysis, Spearman correlations were also used to account for the ordinal nature of PRO-2 items which had no impact on the effect sizes (differences ≤ 0.02; data not shown).

## 3. Results

### Patient characteristics

The study included 684 patients with CD and 548 with UC, the demographics and details of which are summarized in Table 4.

**Table 4.** Summary table of study participants.

		CD (*n* = 684)	UC (*n* = 548)
**Sex**	Female	297 (45.8%)	201 (36.7%)
	Male	208 (32.1%)	207 (37.8%)
	Not known	179 (22.1%)	140 (25.5%)
**Median age in years (IQR)**		39.8 (28.2-54.1)	41.3 (31.8-57.3)
**Ethnicity**	White	441 (68.0%)	363 (66.3%)
	Black	5 (0.8%)	2 (0.4%)
	Asian	25 (3.9%)	17 (3.1%)
	Mixed	8 (1.2%)	11 (2.0%)
	Other	6 (0.9%)	6 (1.1%)
	Not known	163 (25.2%)	159 (29.0%)
**Median years since diagnosis (IQR)**		5.3 (0.6-13.7)	4.4 (1.4-11.4)
**Median age at diagnosis (IQR)**		30.2 (21.6-46.2)	32.2 (24.4-44.4)
**CD Montreal location**	L1	107 (16.5%)	NA
	L2	125 (19.3%)	
	L3	120 (18.5%)	
	L4	8 (1.2%)	
	Not known	288 (44.4%)	
**CD Montreal behavior**	B1	311 (48.0%)	NA
	B2	119 (18.4%)	
	B3	17 (2.6%)	
	Not known	201 (31.0%)	
**CD perianal disease**	No	364 (56.2%)	NA
	Yes	72 (11.1%)	
	Not known	212 (32.7%)	
**UC Montreal extent**	E1	NA	54 (9.9%)
	E2		214 (39.1%)
	E3		92 (16.8%)
	Notknown		188 (34.3%)

Abbreviations: CD, Crohn’s disease; IQR, Interquartile range; UC, ulcerative colitis.

### Clinical disease activity at baseline assessment measured by 4-day PRO-2 and rapid PRO-2

A minimum of 2 days PRO-2 data entry was required to calculate disease activity at baseline, with 933 of 1232 (75.7%) participants completing all 4 days. There was no significant difference in PRO-2 scores between those who completed the rapid PRO-2 and those who completed the full 4-day PRO-2, for any item (all *P* > .05). The proportion of patients categorized as having clinically active or inactive disease remained broadly similar between the 2 scoring methods (Figure 2). For CD, active disease was identified in 442 patients (64.6%) using the 4-day PRO-2 and 458 (67.0%) using the rapid PRO-2; inactive disease was identified in 242 (35.4%) and 226 (33.0%), respectively. For UC, active disease was identified in 317 patients (57.8%) using the 4-day PRO-2 and 359 (65.5%) using the rapid PRO-2; inactive disease was identified in 231 patients (42.2%) and 189 patients (34.5%), respectively. Substantial agreement was seen between the 2 scoring methods (Cohen’s kappa = 0.715 and 0.762 for CD and UC, respectively; *P* < .001 for both). Importantly, results were consistent when analyses were restricted to participants with complete 4-day diary data (removing all participants with < 4 days complete), with substantial agreement again observed between the rapid and 4-day PRO-2 for both CD (Cohen’s kappa = 0.714) and UC (Cohen’s kappa = 0.762; both *P* < .001).

**Figure 2.**
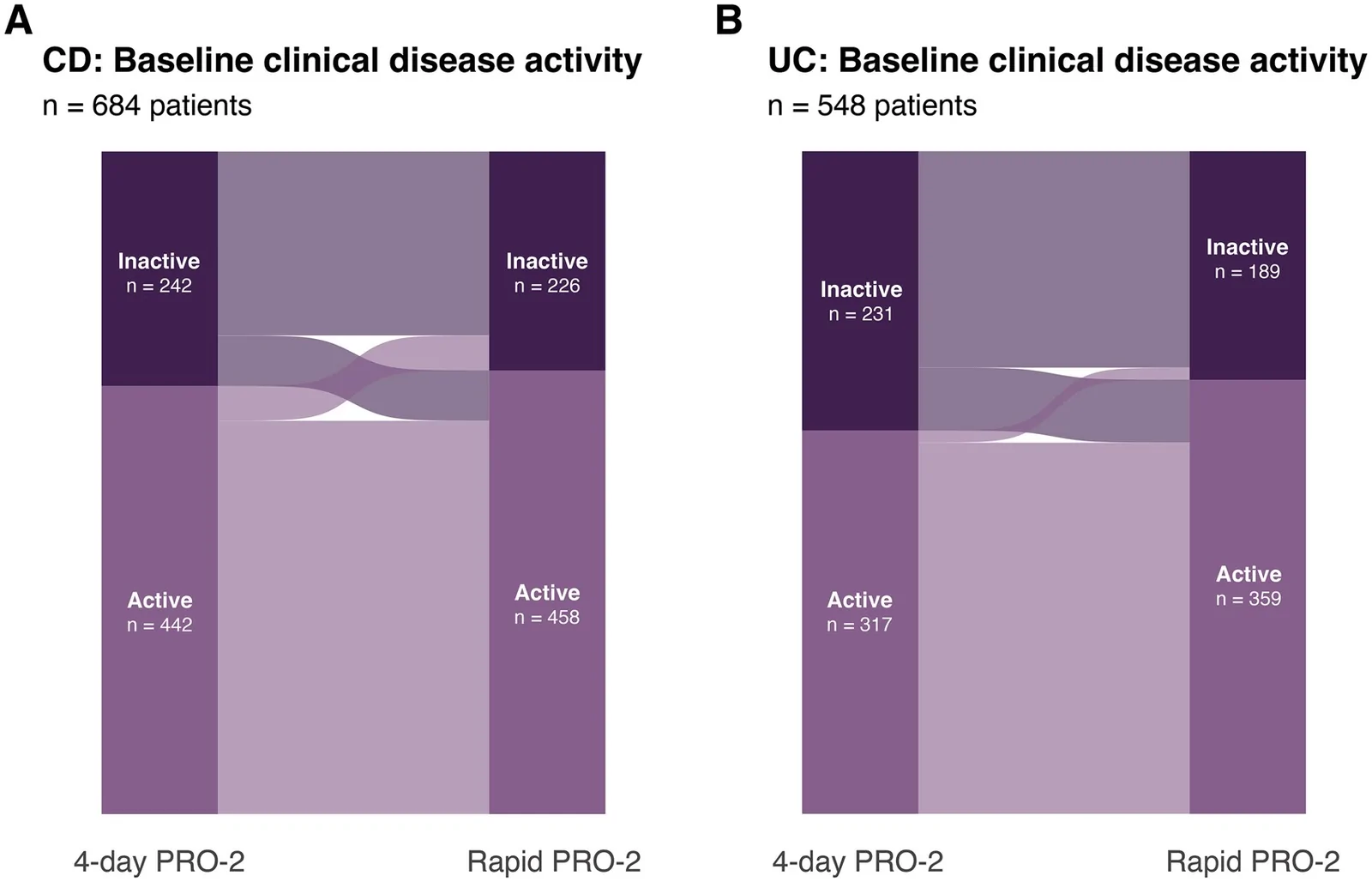
Comparison of baseline clinical disease activity in (A) CD (*n* = 684 patients) and (B) UC (*n* = 548 patients) calculated using either the 4-day PRO-2 score or rapid PRO-2. CD, Crohn’s disease; PRO, patient-reported outcomes; UC, ulcerative colitis.

There was minimal variation on an individual basis, with most patients remaining in the same disease activity category (inactive or active: *n* = 596 [87.1%] for CD and *n* = 486 [88.7%] for UC). Where patients did change disease activity category (*n* = 52 [7.6%] from inactive to active and *n* = 36 [5.3%] from active to inactive for CD and *n* = 52 [9.5%] from inactive to active and *n* = 10 [1.8%] from active to inactive for UC), these were typically patients who were close to the defined PRO-2 thresholds (Figure 3). Where discrepancies were observed in the individual components of the PRO-2 questionnaires used to define disease activity (ie, stool frequency and abdominal pain for CD and stool frequency and rectal bleeding for UC), they typically involved a shift to an adjacent score category (median of 93.6% across CD abdominal pain and stool frequency scores, and median of 93.5% across UC rectal bleeding and stool frequency scores) rather than shifting across multiple individual score categories (Figure 3).

**Figure 3.**
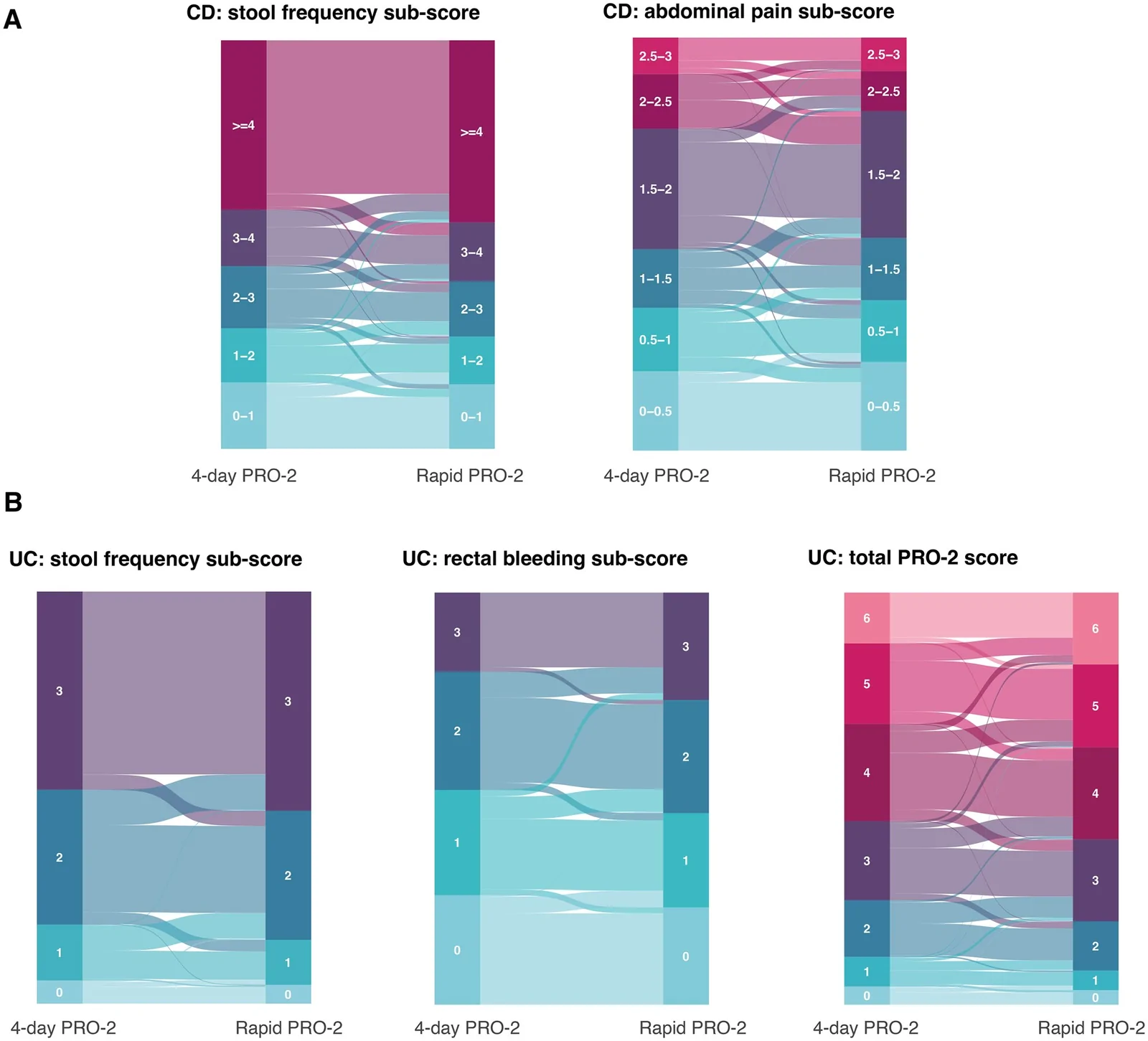
Items of the PRO-2 that are used to calculate disease activity at baseline for (A) patients with CD disease and (B) patients with UC, calculated using either the day 4-day PRO-2 score or the rapid PRO-2 score. Sub-scores were rounded to the nearest whole number, and include the total PRO-2 score for UC patients to reflect how the PRO-2 is calculated. CD, Crohn’s disease; PRO, patient-reported outcomes; UC, ulcerative colitis.

We next sought to identify an explanation as to why patients might shift clinical disease activity groups based on the 2 scoring methods. Based on a binomial logistic regression model, CD patients who had a more variable abdominal pain score across the 4 days were significantly more likely to change groups between the 2 scoring methods (adjusted OR 5.0, 95% CI 3.65–6.85, *P* < .001). UC patients who had a more variable rectal bleeding score across the 4 days were significantly more likely to change groups between the 2 scoring methods (adjusted OR 7.8, 95% CI 5.6–11.0, *P* < .001). Variable stool frequency scores were not significantly associated with a change in groups between the 2 scoring methods for either CD or UC patients (both *P* > .05).

As this study evaluates agreement between PRO-2 measurement approaches, analyses were restricted to within-instrument comparisons and did not include evaluation of PRO-2 against inflammatory biomarkers of disease activity such as CRP and fecal calprotectin.

### Clinical response and remission rates at week 14 measured by 4-day PRO-2 and rapid PRO-2

Of the 1232 participants, only those with clinically active disease at baseline were for follow-up at week 14. Week 14 data were available for 484 participants who met the disease activity threshold, and were subsequently enrolled in IBD-RESPONSE. Similar trends to baseline were observed at week 14 post-treatment commencement when evaluating response to an advanced therapy. The proportion of patients classified as non-response, response or remission was similar between both scoring methods (Figure 4). For CD, remission was identified in 102 patients (40.5%) using the 4-day PRO-2 and 109 (43.3%) using the rapid PRO-2; response was identified in 63 patients (25.0%) and 59 patients (23.4%); and non-response was identified in 87 patients (34.5%) and 84 patients (33.3%), respectively. For UC, remission was identified in 101 patients (43.5%) using the 4-day PRO-2 and 93 (40.1%) using the rapid PRO-2; response was identified in 74 patients (31.9%) and 77 patients (33.2%); and non-response was identified in 57 patients (24.6%) and 62 patients (26.7%), respectively. Again, substantial agreement was observed between the 2 scoring methods (Cohen’s kappa = 0.653 and 0.743 for CD and UC, respectively; both *P* < .001). This remained true even when analyses were restricted to participants with complete 4-day diary data (removing all participants with < 4 days complete), with substantial agreement again observed between the rapid and 4-day PRO-2 for both CD (Cohen’s kappa = 0.669) and UC (Cohen’s kappa = 0.754; both *P* < .001).

**Figure 4.**
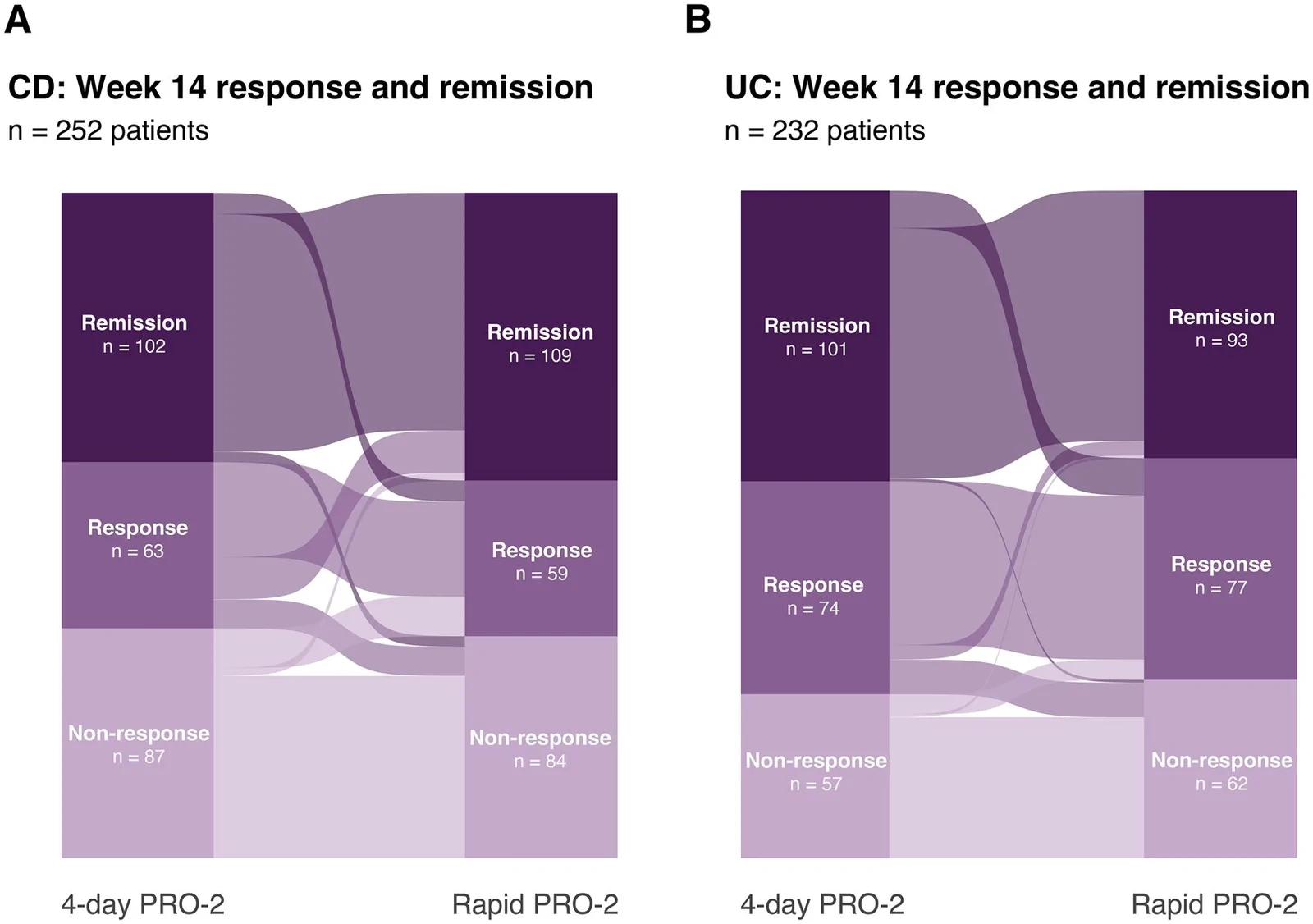
Clinical response and remission to treatment at week 14 for (A) patients with CD and (B) patients with UC, calculated using either the 4-day PRO-2 score or the rapid PRO-2 score. CD, Crohn’s disease; PRO, patient-reported outcomes; UC, ulcerative colitis.

On an individual basis, *n* = 195 (77.3%) CD patients were classified in the same category based on both scoring methods (Figure 4). Of the 57 discrepancies observed (23%), 33 (57.9%) involved a shift between remission/response and non-response with 26 patients moving between response and non-response and just 7 moving between remission and non-response. The remaining 24 discrepancies (42.1%) reflected shifts between response and remission.

Most UC patients were also classified into the same category based on both scoring methods (*n* = 193 [83.1%]; Figure 4). Of the 39 discrepancies observed (17%), 21 (53.8%) involved a shift between remission/response and non-response with 19 patients moving between response and non-response and just 2 moving between remission and non-response. The remaining 18 discrepancies (46.2%) reflected shifts between response and remission.

The underlying individual components of the PRO-2 questionnaires used to calculate response and remission showed similar findings to baseline. Where discrepancies were observed, they typically involved a shift to an adjacent score category (median of 94.8% across CD abdominal pain and stool frequency scores, and median of 97.0% across UC rectal bleeding and stool frequency scores) rather than shifting across multiple individual score categories (Figure 5).

**Figure 5.**
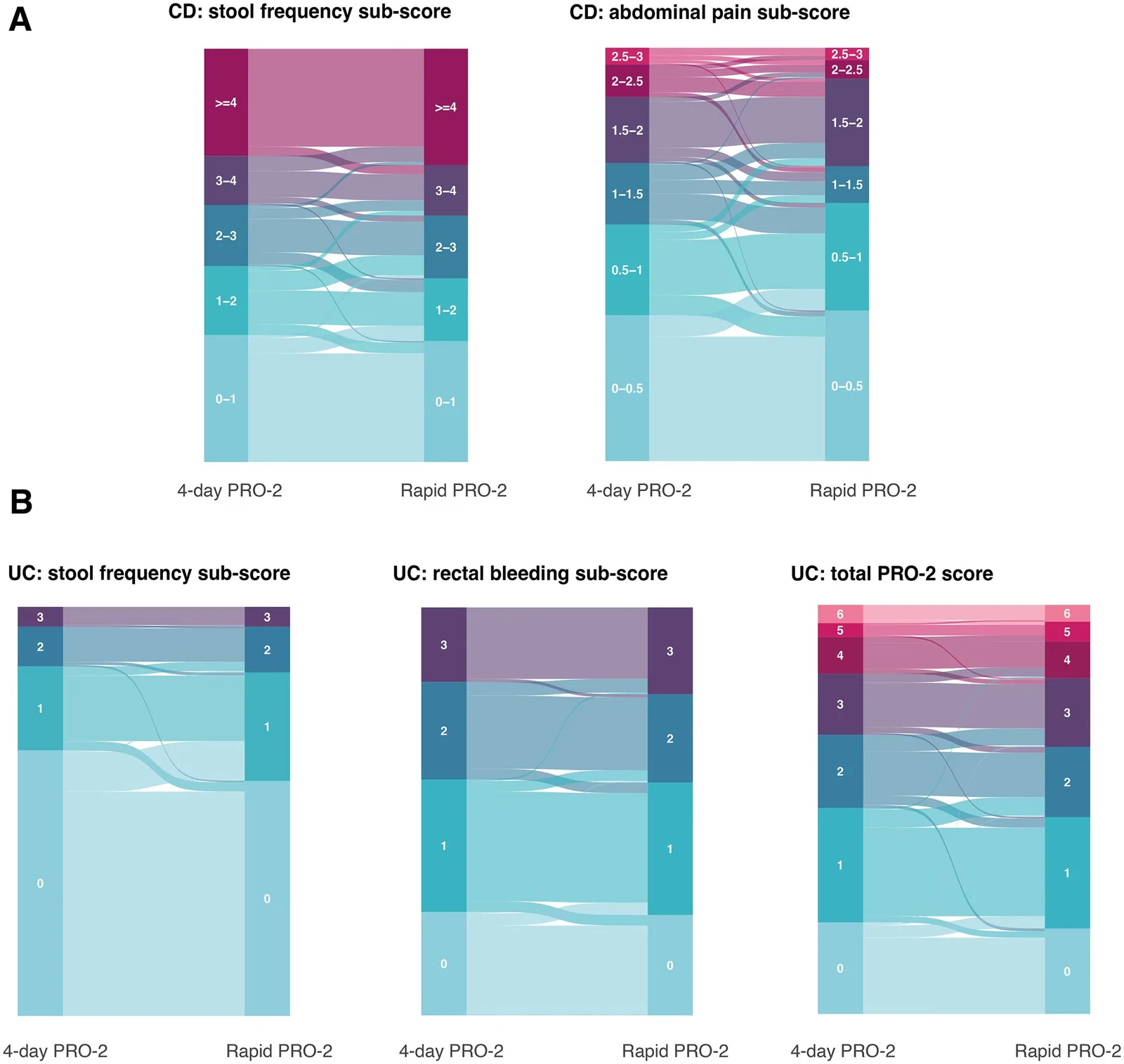
Items of the PRO-2 used to calculate clinical response and remission at week 14 following treatment for (A) patients with CD and (B) patients with UC, calculated using either the 4-day PRO-2 score or the rapid PRO-2 score. Sub-scores were rounded to the nearest whole number, and include the total PRO-2 score for UC patients to reflect how the PRO-2 is calculated. CD, Crohn’s disease; PRO, patient-reported outcomes; UC, ulcerative colitis.

### Responsiveness of the rapid PRO-2 vs 4-day PRO-2 to clinical change

The responsiveness of the rapid PRO-2 and 4-day PRO-2 diaries to clinical change was assessed using SRMs and adjusted effect sizes for each individual PRO-2 item in both CD and UC, as well as the total score in UC only (Tables 5 and 6). Negative effect sizes indicate improvement in symptoms between baseline and week 14.

**Table 5.** SRM and adjusted effect size for individual PRO-2 items measured using rapid PRO-2 and 4-day PRO-2 diaries in CD.

Item	SRM		Adjusted effect size	
	**Rapid PRO-2**	**4-day PRO-2**	**Rapid PRO-2**	**4-day PRO-2**
**Stool frequency**	−0.640	−0.639	−0.561	−0.607
**Abdominal pain**	−0.879	−0.951	−0.820	−0.910

Abbreviations: CD, Crohn’s disease; PRO, patient-reported outcomes; UC, ulcerative colitis.

**Table 6.** SRM and adjusted effect size for individual PRO-2 items and total score measured using rapid PRO-2 and 4-day PRO-2 diaries in UC.

Item	SRM		Adjusted effect size	
	**Rapid PRO-2**	**4-day PRO-2**	**Rapid PRO-2**	**4-day PRO-2**
**Stool frequency**	−1.008	−1.043	−0.826	−0.852
**Rectal bleeding**	−1.183	−1.225	−0.901	−0.966
**Total PRO-2**	−1.339	−1.300	−1.043	−1.028

Abbreviations: PRO, patient-reported outcomes; UC, ulcerative colitis.

Across both CD and UC, responsiveness patterns were consistent between SRM and adjusted effect sizes, with most values falling within the moderate to large effect size range based on Cohen’s thresholds. In CD, abdominal pain demonstrated the highest responsiveness, while in UC, rectal bleeding was the most responsive item. Although the 4-day PRO-2 diary generally yielded slightly higher responsiveness estimates, the rapid PRO-2 showed comparable responsiveness across all items and total scores, supporting its utility as a practical alternative.

## 4. Discussion

Participant burden and “questionnaire fatigue” represent major barriers to patient retention in research which can be compounded by longitudinal data collection at multiple time points. This methodological analysis aimed to evaluate whether a condensed 2-day symptom diary collected in a single day (rapid PRO-2) could serve as a viable alternative to a 4-day PRO-2, used in the IBD-RESPONSE study.^1^ Our findings lead us to conclude that a 2-day PRO-2 score that is collected in a single day, may be a viable alternative to longer diaries, particularly in the case of observational studies where the primary aim is to distinguish treatment benefit from non-benefit rather than precisely quantify exact remission rates. The rapid PRO-2 showed limited difference to a prospectively collected 4-day PRO-2, suggesting that essential symptom data is retained. This approach aligns with a need for streamlined PROs and patient-centered care in longitudinal research. Notably, collecting a 2-day score in a single day also allows for an average to be taken and therefore enhances the robustness of data for analysis, compared to collecting a single-day score.

At week 14, despite there being substantial agreement between the rapid PRO-2 and the 4-day PRO-2, 23% of patients with CD and 17% of patients with UC were assigned different response categories. Importantly, most discrepancies involved shifts to adjacent categories, for example, between response and remission. Reassuringly only a small proportion of patients (10% CD, 8% UC) were reclassified between response and non-response. Additionally, there was a very small number of instances of re-classification between remission and non-response (*n* = 7 for CD, *n* = 2 for UC). These cases likely reflect a small subset of patients experiencing highly fluctuating symptoms. It is likely that some individuals exhibit a more dynamic nature of symptoms, and these findings highlight the inherent challenge of reliably capturing and categorizing symptoms of that nature for those patients through any PRO method, regardless of diary length. This symptomatic dynamism highlights the value of complementing PROs with objective markers of luminal inflammation, for example, biochemical markers including fecal calprotectin and C-reactive protein, mucosal visualization at endoscopy or histological evaluation of biopsy samples.

In addition to comparing disease categorization at baseline and week 14, we examined how well the rapid PRO-2 captured clinical change between the 2 timepoints, which given its shorter duration could be subject to more influence from noise that is, variation in symptoms across days. The rapid 2-day PRO-2 demonstrated comparable responsiveness to the 4-day PRO-2 across all individual items for both CD and UC and total scores for UC, supporting its use as a practical alternative for capturing clinical change longitudinally. Although the 4-day diary showed slightly higher responsiveness estimates in some measures, the differences were small and therefore unlikely to be clinically meaningful. Adjusting for the correlation between baseline and week 14 values within individuals did not alter these conclusions, underscoring the robustness of the responsiveness findings. These results suggest that shortening symptom diaries can reduce patient burden without compromising the ability to detect meaningful clinical changes.

While the body of literature on patient reported outcomes in IBD research is expanding, methodological details surrounding the collection of this data, such as whether data has been collected retrospectively or prospectively and the duration over which data is collected, remain inconsistently reported. For UC, the PRO-2 is already typically collected across 3-days.^16^^,^^17^ There are also examples where a 3-day retrospective PRO-2 is assessed.^18^ Despite the suggested 7-day period of PRO-2 data collection for CD, studies such as Hoekman et al. have already adopted more pragmatic approaches, including patients with up to 4 days of missing diary entries.^19^ Although these studies still use a longer collection window than our proposed rapid PRO-2, they reflect a growing recognition of the real-world challenges in lengthy symptom tracking for patients and support the feasibility of shorter instruments. Indeed, Henao et al. found patients to have relatively little variability in bowel symptoms across a 7-day period, allowing for an accurate computation of a short CDAI and 6-point Mayo score using just 1–3 days of data.^20^ Furthermore, patients were found to have good recall of bowel symptoms for up to 8 days, supporting the use of a retrospective measure of symptoms.^20^

A key strength of this study is its pragmatic design within a large, multicenter prospective cohort, allowing assessment of symptom reporting under real-world conditions. By directly comparing the rapid PRO-2 to a 4-day PRO-2, this work provides evidence to support approaches that may reduce participant burden in longitudinal IBD studies. A limitation of our study is the lack of validation of the rapid PRO-2 against the full 7-day standard for CD. However, given the trends in the literature and our findings against a 4-day PRO-2, the rapid PRO-2 is likely to offer a meaningful and pragmatic tool for clinical research, particularly in studies of a longitudinal nature where participant burden is a key concern. In addition, the inclusion of a single retrospective day introduces a potential for recall bias. However, given the short recall window and the nature of acute symptom reporting, this is likely to be minimal. While widely used, the PRO-2 still only captures part of the patient experience. The instrument was derived from the clinician-developed Crohn’s Disease Activity Index (CDAI) rather than through direct patient input and therefore may not fully reflect the patient perspective on disease burden (eg, by excluding extraintestinal symptoms). This study did not assess the relationship between PRO-2 and objective markers of inflammation (eg, endoscopy, fecal calprotectin or CRP), as the aim was to evaluate methodological agreement between duration of PRO-2 rather than validity of each criterion in measuring inflammatory disease activity. Finally, while the simplified approach enhances feasibility, it may not fully capture day-to-day variability in disease activity. Small shifts between non-response/response/remission categories observed in a minority of patients could have greater implications in interventional clinical trials which could impact the conclusions drawn about therapeutic efficacy.

Recognizing the need for balance between methodological rigor and pragmatic feasibility in the design and use of PRO measures, our data leads us to conclude that a shorter PRO-2 provides a practical balance between data accuracy and participant compliance. By reducing patient burden, future studies may be able to enhance participation and retention, support reproducibility and so contribute to advancing patient-centered research in IBD.

## Data Availability

The IBD-RESPONSE clinical metadata presented in this manuscript is available via managed access to ensure data is used in line with the consent provided by the IBD-RESPONSE research participants. Requests for data access should be directed to ibd.response@newcastle.ac.uk.
